# Measuring the Benefits of a Regional Imaging Environment

**DOI:** 10.1007/s10278-017-9963-8

**Published:** 2017-03-15

**Authors:** Jason Nagels, David Macdonald, Christine Coz

**Affiliations:** HDIRS, 7100 Woodbine Ave, Suite #214, Markham, ON L3R5J2 Canada

**Keywords:** PACS, Health information exchange (HIE), Digital imaging and communications in medicine (DICOM), Enterprise PACS, Foreign exam management (FEM)

## Abstract

The use of central diagnostic imaging repositories (DIRs), that allow separate organizations with disparate PACS systems to seamlessly share patient data, is becoming more common; and as a result, the documentation of measurable benefits is a key deliverable to all stakeholders. Central DIRs and the implementation of *foreign exam management (FEM)* provide clinical users with the ability to seamlessly access DI exams and reports that originate from an outside location. FEM has been implemented to varying degrees across regional DIRs within Canada [1]. Historically, measuring the benefits of transitioning from a film-based environment to a PACS environment has been documented as being difficult and poses challenges [2]. Many of these same challenges are exacerbated when trying to measure benefits across a regional DIR. From the DIR, it is easy to report on the overall number of foreign exams that were transferred from the DIR to each individual site. While this metric does provide some insight into the number of patients migrating between hospitals and clinics, and demonstrates a growth pattern of the ingestion of foreign exams, it does not provide insight into the use and value of these foreign exams to the clinical user. At the outset, we hypothesized that quantifiable benefits could be measured, but would likely yield understated measurable results, due to the complexities involved in gathering data. In spite of this challenge, with targeted analysis across the region, together with many qualitative results from clinical users, a compelling picture would emerge.

## Background

A key initiative that Canada Health Infoway (CHI) set out to accomplish is the goal of “One Patient, One Record.” Part of this goal involves allowing clinical users to *seamlessly* access diagnostic imaging (DI) exams that did not originate at their local institution. In the province of Ontario, a government agency, eHealth Ontario, oversaw the creation of 4 DIRs. This paper focuses on the benefits analysis of one specific DIR, Hospital Diagnostic Imaging Repository Services (HDIRS), which manages the DIR for sites in the Toronto-East region of Ontario.

The DIR is setup as a hub and spoke environment. The individual connected organizations are spoke sites that archive to the hub DIR. HDIRS has over 100 individual sites contributing DI exams to the DIR, resulting in approximately 4,800,000 exams a year. The seamless access of DI exams is accomplished by connecting individual hospitals and clinics to central diagnostic imaging repositories (DIRs), and allowing the sites to consume foreign exams.

In 2013, Canada Health Infoway Standards Collaborative Working Group 10 defined *foreign exam management as,* “*An instance of a radiology exam with images and/or reports that were originally acquired outside of the local enterpri*se” [3]. In a typical hospital/clinic environment, a site’s local PACS *only* has access to diagnostic images that were acquired at that local site, and only healthcare providers who share the *same* PACS system have access to these exams. Alternatively, within a DIR environment, when foreign exam management is enabled, authorized users are able to access images and/or reports that were originally acquired *outside* their local PACS in a way that is consistent with accessing images that were acquired locally [4].

Initial attempts to measure the advantages of seamless access to foreign exams through a DIR were met with challenges. For example, a site’s local PACS logs may not natively discriminate the access of foreign exams vs. local exams. In order to achieve detailed logs on the access of foreign exams, custom reports were required. Additionally, in order to document a reduction in re-imaging patients as a result of having access to a foreign exam, which was one of the primary benefits expected at the outset of this project, documentation of a decision not to place an order for an exam would be required. Such information cannot be collected via any currently known automated means.

Despite the value in qualitative feedback, HDIRS’s funders and stakeholders require a deeper analysis of quantitative data that can be measured to represent concrete benefits.

## Methods

In order to capture quantitative measurable benefits, three specific questions were reviewed and studied. The questions are (1) how often are foreign exams accessed at a local site? (2) has there been a reduction in the number of CD imports for participating sites? and (3) are there any methods that can be implemented to measure a reduction in reimaging patients, due to the availably of foreign exams?

The following describes the methods implemented to answer each of these questions:

1. *Measure the rate at which clinical users are accessing foreign exams retrieved into their local PACS:*

**Fig. 1:**
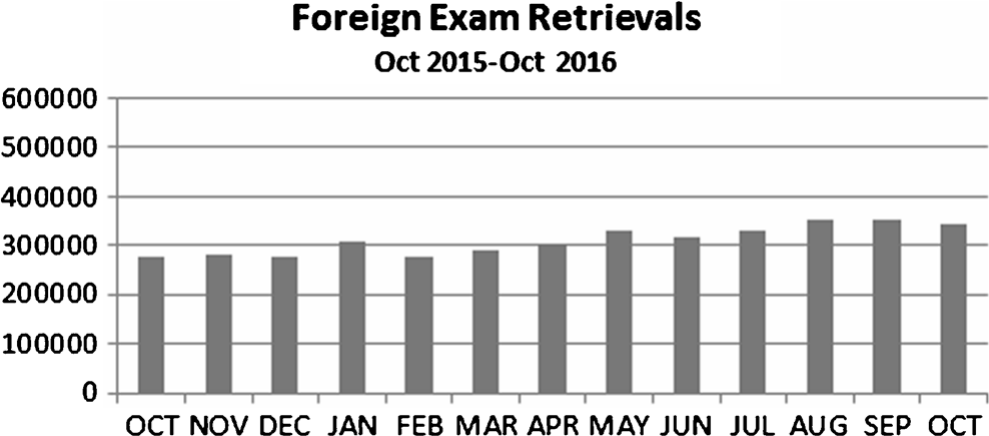
Foreign exam retrievals

There are currently 60 individual sites that have the ability to seamlessly retrieve foreign exams from the DIR directly into their local PACS. Clinical users from these sites have grown to expect access to foreign exams, allowing the user to view a patient’s complete longitudinal DI record. The number of foreign exams transferred from the DIR to the individual sites (Fig. 1), provides insight into the steady and growing need of a site’s ability to access outside imaging. Currently, the consuming sites are trending towards an average of just over 300,000 foreign exams retrieved every month. This particular metric demonstrates that patient care is not confined to one physical location, and in order optimize patient care, clinical users require access to the longitudinal history of the patient. However, this metric does not provide any insight into how these exams are accessed, and what role outside imaging plays in the context of providing patient care.

As a first step towards gaining insight into the relationship between a patient’s local imaging and their longitudinal DI history, an analysis of exams in the DIR was conducted. The analysis looked at 12 months of data and compared how many unique patients received a local DI exam compared to the number of foreign exams that patients had available in the past 1 year and 3 years. This measurement would demonstrate the rate of availability between local imaging and foreign prior exams.

In order to analyze how the foreign exams are used in context of patient care, logs and auditing activity are required at a local site level. The main challenge with obtaining this information is that typically a PACS is not designed with the ability to distinguish foreign exams from those locally created. Additionally, the detail that various PACS vendors are able to capture varies. Therefore, the ability to measure the number of foreign exams accessed at local sites varies depending on the capabilities of each PACS vendor. Some vendors are able to report only a total number of foreign exams accessed, whereas other vendors are able to report on more specific details such as which users accessed the exams, number of local exams accessed, and number foreign exams accessed. The desired result of analyzing the custom audits is to see if we can measure a rate of relation between patient’s receiving local imaging, and how often foreign exams are accessed.

The information in the custom audits allows us to see how many patients were locally imaged that had available foreign exams and compare how often foreign exams for these patients are accessed when available.

2. *Measure if there has been an impact on local site’s reliance on the use of importing images through physical media:*

Historically, as organizations transitioned from a film-based imaging environment, to a PACS environment, sites have relied on the ability to share patient’s DI exams via physical media, such as a CD or DVD [5]. There is an expectation that network transfer of images will eventually replace the transfer of physical media as a means of sharing patient’s data [6]. This benefits measurement is based on the assumption that as sites are able to access foreign imaging through the DIR, there will be a measurable reduction on imported outside physical media.

HDIRS member sites were provided access to outside imaging in two separate phases. *Phase 1* was the ability to access DI exams on the DIR using a regional DIR viewer. The viewer is separate from their local PACS and requires the user to login with unique authentication. *Phase 2* was the ability for sites to consume foreign exams directly into their local PACS through FEM ingestion. Our hypothesis is that we would see a slight reduced number of CD/DVD imports during phase 1, and the number of CD/DVD imports would dramatically drop during phase 2.

HDIRS member sites have a standard in place that ensures anytime a site imports an outside CD/DVD they will create a local order for the exam that has a study description of “IMPORT,” “OUTSIDE,” or “REFERENCE.” A 4-year review of exams received by the sites was conducted, to look at the number of exams with the name “IMPORT,” “OUTSIDE,” or “REFERENCE” in the study description. This study compares the 3 years before the sites had access to ingest foreign exams (phase 1) compared to the fourth year in which the sites went live with FEM (phase 2).

3. *Conduct a focused study over an isolated timeframe to determine if a measureable reduction in re-imaging patients has occurred due to the availability of foreign exams:*

Studies have shown that importing outside imaging into a local PACS reduces the rate of repeat imaging to the patient [7]. In a regional environment such as HDIRS, it is expected that the availability of a patient’s longitudinal record will have a similar effect; however, this expectation can be a challenge to quantify as it involves measuring when something does not occur. Survey results have been used to capture the clinical user’s impression of a reduction in repeat imaging; however, these impressions for the most part are anecdotal and qualitative.

One of the sites connected to HDIRS has implemented the following workflow in the emergency room (ER):

- Patient is ordered to receive imaging,
- The technologist will manually check to see if the patient has any potential foreign imaging that could overlap with the ordered procedure,
- If overlapping/duplicate imaging exists, the technologist will notify the ordering physician,
- The ordering physician will decide if the foreign imaging is sufficient to prevent the patient from receiving the ordered procedure.

Two separate 2-week studies were conducted, one completed in September 2014 and another in September 2015 to determine how many patients in that time frame were prevented from *‘*repeat imaging. The staff was provided a calendar covering the 2-week time period and would note when a patient was prevented from being locally imaged, due to the availability of foreign exams.

The intention of the study was to capture the following: (1) how many patients were marked as prevented from re-imaging, (2) how many patients received a DI exam in the ER during the time of the study, and (3) how many of the total patients that received DI exams had available foreign exams.

At the end of the 2 weeks, the departments provided the total number of patients were prevented from repeat imaging. Additionally, the site provided a list of the total number of patient’s that received local imaging in ER during that period of time. The total number of patients that received imaging was referenced against to the DIR to display how many of those patients had foreign imaging available in the DIR.

The study demonstrated the rate of preventing repeat imaging in the ER for this site when a patient has foreign exams available.

## Results/Evaluation

1. Measure the rate at which clinical users are accessing foreign exams retrieved into their local PACS:

A review of available foreign exams for a community hospital was conducted between the months of April 2015 and March 2016. The review displayed the total numbers of “unique patients” that received a DI exam for each month. The “unique local patients” were compared against how many foreign exams were available for the patient within the past year and past 3 years.

From the chart below (see Table 1), a patient will have foreign imaging available that was performed within the past year on average about 28% of the time, and on average, 41% of the time foreign imaging that occurred within the past 3 years is available.

**Table 1:** Availability of foreign exams within the past year and previous 3 years at a community hospital

Month	Hospital patients that received diagnostic imaging	Number of DI patients with available foreign exams in the past year	Percentage of available foreign exams within the past year	Number of available foreign exams within the last 3 years	Percentage of available foreign exams within the past 3 years
201,603	254,005	73,580	29.0%	109,282	43.0%
201,602	236,357	69,170	29.3%	101,695	43.0%
201,601	241,226	70,540	29.2%	104,218	43.2%
201,512	224,865	65,435	29.1%	96,553	42.9%
201,511	250,477	71,305	28.5%	105,843	42.3%
201,510	250,981	71,532	28.5%	105,559	42.1%
201,509	244,805	67,879	27.7%	100,297	41.0%
201,508	230,524	63,588	27.6%	94,016	40.8%
201,507	247,517	67,737	27.4%	100,239	40.5%
201,506	262,833	70,556	26.8%	104,943	39.9%
201,505	253,950	67,008	26.4%	100,040	39.4%
201,504	252,153	65,795	26.1%	98,868	39.2%

In order to understand what the rate of access for foreign imaging is, custom auditing was setup and configured for one the contributing PACS vendors. The custom auditing was implemented at three unique hospital organizations. The details below represent the following: (1) Of the patients that received local imaging at a hospital, how many had foreign exams available, and (2) how many local patients had foreign exams accessed.

For the purpose of this paper, an “accessed” foreign exam is defined as an exam that either the images or the report has been viewed by a PACS users (see Table 2).

**Table 2:** Access of locally treated patients with available foreign exams

Site	Number of local patients with available foreign exams	Number of patients with foreign exams accessed	Percentage of patients with accessed foreign exams
Downtown Hospital (11 month average)	3798	1799	47.37%
Community Hospital (11 month average)	4259	1139	26.74%
Regional Health Centre (10 month average)	2539	515	20.28%

The values in this chart demonstrate consistent use of a patient’s longitudinal DI record. These results indicate that the access of a patient’s outside imaging has become a regular expected part of the clinical user’s day to day workflow. A conclusion can be drawn that access to foreign exams is positively benefitting the patient care that clinical users are able to provide.

**Fig. 2:**
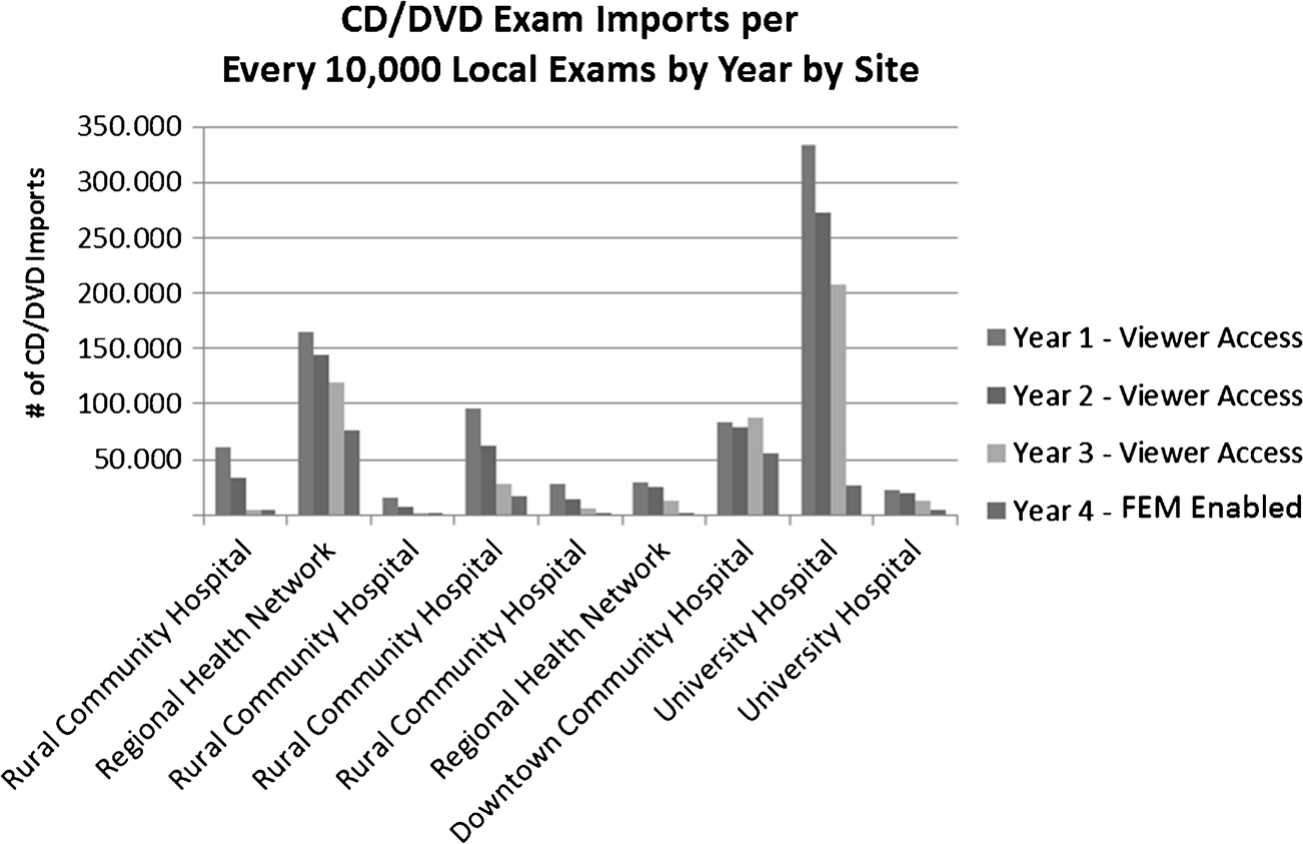
CD/DVD exam imports

2. Measure if there has been an impact on local site’s reliance on the use of importing images through physical media:

The graph below (see Fig. 2) demonstrates *the number of CD/DVD exam imports per every 10,000 local exams* by year by site. This chart represents a wide range of different organization types that include two university hospitals, four rural community hospitals, two regional health networks, and a downtown community hospital.

The results illustrate that there was an immediate reduction in the number of CD/DVD imports handled by a site, based on availability to the DIR viewer. The year the sites enabled FEM there was an even greater reduction in the number of of exams with the name “IMPORT,” “OUTSIDE,” or “REFERENCE” in the study description.

**Fig. 3:**
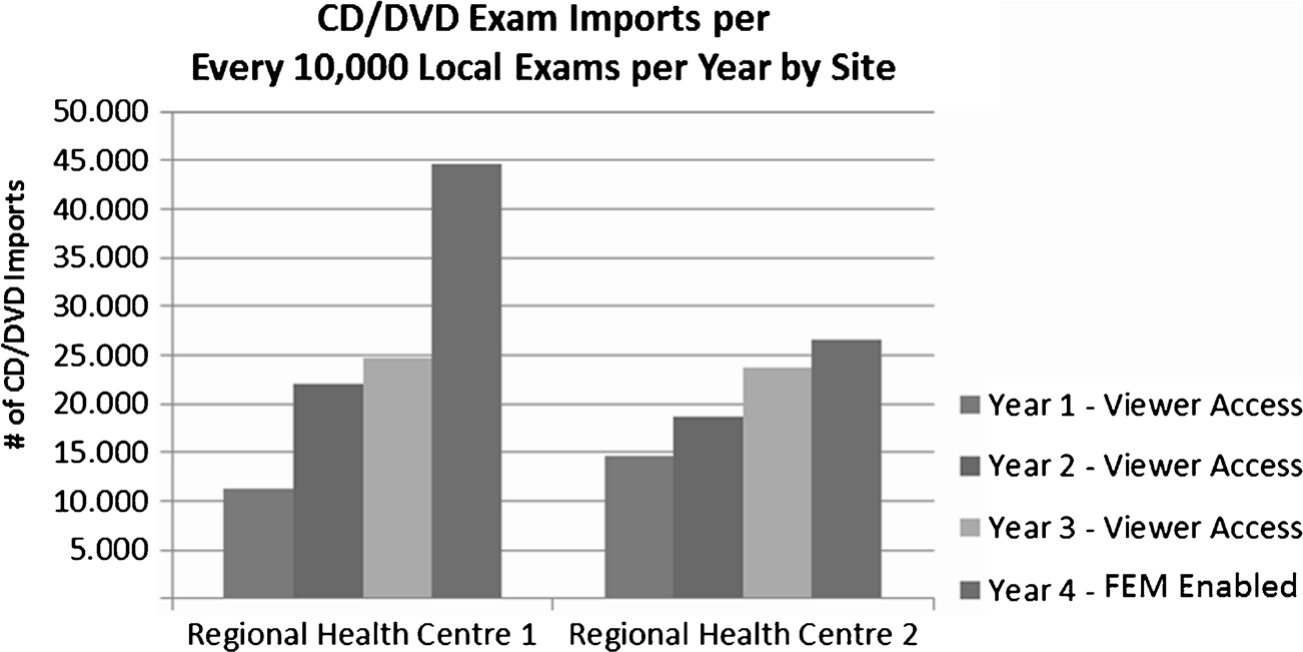
Regional health centers that have sharing patterns between sites that are not currently connected to HDIRS

It should be noted that there are outlier sites that did not see a reduction of imports. The chart below (see Fig. 3) shows two regional health centers that have sharing patterns between sites that are not currently connected to HDIRS, and therefore still heavily rely on importing patient data from these sites.

Overall, the reduction in CD/DVD imports to the majority of the sites represents a savings in the cost of resource time and a significant improvement in the seamless access of a patient’s longitudinal DI exams.

3. Conduct a focused study over an isolated timeframe to determine if a measureable reduction in re-imaging patients has occurred due to the availability of foreign exams:

Two separate studies were conducted, in 2014 and 2015, to see if access to outside imaging in the ER of a regional health network had any impact on reducing re-imaging patients. As per the steps detailed in the methods section above, the hospital staff manually marked off in a tally sheet when a patient was prevented from repeat imaging.

The results of the study are demonstrated below (see Table 3).

**Table 3:** Prevention of repeat imaging based on available foreign exams

Date	September 14–27 2015	September 13–26, 2015
Total number of ER patients that received Diagnostic Imaging	894	880
Total number of imaged patients with foreign imaging available	36	34
Percentage of patient’s prevented from repeat imaging	17%	15%

These results correspond with the expectation that availability to outside imaging can prevent the rate of repeat imaging. This study was captured against one department across a four-site health network. If these studies from 2014 and 2015 are extrapolated across all HDIRS sites, it would mean that thousands of unnecessary exams are being avoided annually. Extended further, this represents a potential province wide benefit equivalent to millions of dollars each year. In parallel to the study, an HDIRS survey distributed in 2015, documented that 30% of physicians responded that access to the DIR has reduced the number of duplicate studies ordered.

## Conclusion

The results provide an indication of accrued benefits of FEM through a regional DIR. The pattern and rate of access to foreign exams demonstrate steady growth and consistent use. The results reveal that providing seamless access of foreign exams directly into a local PACS has improved access to a patient’s longitudinal record and has mitigated the concern of accessing exams from physical media. Additionally, the “repeat imaging” study conducted validates that the access of foreign exams has prevented patients from repeat imaging.

The studies in this paper are a snapshot of the overall benefits of a regional DIR. There are many areas that qualitatively have been shown to yield clinical benefits, such as improved patient outcomes, reduced time to treatment, and increased confidence in clinical decision making to name a few. This paper provides a view of three quantitative measurements that indicate the benefits of a regional imaging environment.
